# Exploring the colloidal stability of curcumin nanoparticles formed by nanoprecipitation

**DOI:** 10.1016/j.crfs.2025.101275

**Published:** 2025-12-11

**Authors:** Nicolas Didat, Jordane Jasniewski, Pierrick Durand, Sabine Bouguet-Bonnet, Younes Bouizi, Florentin Michaux

**Affiliations:** a Université de Lorraine, LIBio, Nancy, F-54000, France; b Université de Lorraine, CNRS, CRM2, Nancy, F-54000, France

**Keywords:** Curcumin nanoparticles, Colloidal stability, Ostwald ripening, Antisolvent precipitation, Solubility, Particle growth

## Abstract

Pure curcumin nanoparticles were prepared by antisolvent precipitation using acetone as the solvent and water as the antisolvent, offering a simple and food-compatible strategy to enhance curcumin dispersibility in aqueous environments. The colloidal stability of the aqueous dispersions was assessed by static multiple light scattering. Progressive particle size growth led to sedimentation. This study then focused on the stability of nanoprecipitated curcumin prior to sedimentation, in order to unambiguously determine the mechanisms responsible for destabilization. Particle size evolution over time was monitored by dynamic light scattering. The physical state of curcumin was determined by ^1^H NMR and XRD analyses, confirming that neither coalescence nor crystallization occurred. Ostwald ripening was successfully confirmed by modeling the particle growth kinetics using the Lifshitz-Slyozov-Wagner (LSW) theory. Nevertheless, it is known that the LSW model remains superficial. That is why an original approach is used in this study to validate the destabilization mechanism. This involves varying the composition of the solvent in order to modify the solubility of curcumin in the continuous phase, a key parameter in the context of Ostwald ripening. Thus, varying the acetone content demonstrated the role of curcumin solubility in governing dispersion stability, confirming that Ostwald ripening occurred, as faster particle growth was observed at higher acetone levels. Moreover, curcumin solubility in the continuous phase derived from the LSW model was consistent with measurements performed by HPLC. These findings demonstrated that amorphous curcumin nanoparticles undergo Ostwald ripening in stabilizer-free aqueous systems, with ripening rates controlled by solvent-induced solubility changes.

## Highlights

• Curcumin nanoparticles were prepared by antisolvent precipitation.
• Particle growth was observed over time in aqueous dispersion.
• Growth behavior was modeled using the LSW theory.
• Predicted and experimental solubilities showed strong agreement.
• Nanoparticles exhibited significantly enhanced antioxidant activity compared to native curcumin.

## Graphical abstract

**Figure:**
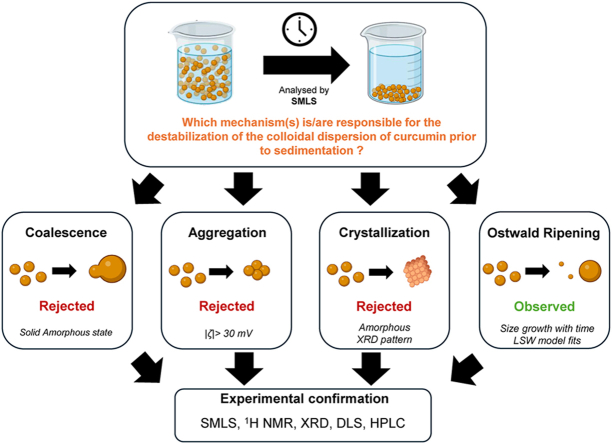

## 1 Introduction

In food systems, many bioactives such as phenolic compounds, vitamins, and essential oils contribute to health benefits, but their practical use is often limited by poor water solubility and instability under processing and storage conditions (Wang et al., 2021). Similar issues are also observed in the pharmaceutical field, where active compounds often face solubility and stability challenges (Amidon et al., 1995). These limitations not only decrease bioavailability but also limit their direct incorporation into food or nutraceutical formulations. To overcome these issues, particle size reduction down to the nano- or microscale has emerged as an effective strategy, as it increases the surface area thereby enhancing the dissolution rate of poorly soluble compounds and improving their functional performance (Peng et al., 2018; Hashemi et al., 2025; Pugazhendhi et al., 2025). Among bioactive food compounds, curcumin, a hydrophobic polyphenol extracted from *Curcuma longa*, stands out due to its wide range of biological activities, including anti-inflammatory, antioxidant, and anticancer effects (Kant et al., 2014; Gunes et al., 2016; Zhong et al., 2024). Despite these promising attributes, the poor water solubility of curcumin and its rapid degradation under certain environmental conditions severely limit its effectiveness in both medicinal and food applications (Wang et al., 2021; Peng et al., 2018; Urošević et al., 2022; Lan et al., 2023). Consequently, numerous studies have focused on developing nano-formulations of curcumin, leading to a wide variety of nano-systems with enhanced performance. However, most of these nanoformulations rely on encapsulation techniques involving carriers such as polymers, lipids or proteins, which can complicate formulation, increase cost, and introduce stability issues of their own (Azad et al., 2024; Can et al., 2025). In contrast, studies focusing on nanoparticles made only of curcumin (without encapsulating materials) are much less common (Yadav and Kumar, 2014; Hettiarachchi et al., 2021; Wu et al., 2023). In general, these strategies can be divided into two main approaches: top-down and bottom-up. Top-down methods rely on mechanically breaking down larger particles but are often limited by high energy requirements, risk of contamination, poor size uniformity and potential degradation of the molecule (Pugazhendhi et al., 2025; Verma et al., 2022). In contrast, bottom-up methods build nanoparticles through processes such as self-assembly, crystallization or precipitation. Among these, nanoprecipitation has gained considerable attention due to its simplicity, cost-effectiveness and scalability (Liu et al., 2020; Silva et al., 2023). Originally introduced by Fessi et al. (1989), this method relies on the rapid mixing of an organic phase containing the active compound with a non-solvent (commonly water), leading to spontaneous nucleation followed by particle growth (Fessi et al., 1989). Nanoprecipitation has been, successfully applied to a variety of food grade bioactive compounds, including lycopene (Anarjan and Jouyban, 2017), quercetin (Jiang et al., 2022), resveratrol (Xing et al., 2022) and curcumin (Yadav and Kumar, 2014) yielding nanoparticles with controlled size, morphology, and improved dispersion stability. Further illustrating the versatility of nanoprecipitation, Thorat and Dalvi (2015) showed that applying ultrasound with stabilizer promoted the formation of orthorhombic crystalline forms of curcumin, whereas raw curcumin or curcumin precipitated without additives crystallized in the monoclinic form. This polymorphic transition is critical as crystalline structure strongly influence solubility, dissolution rate, and overall bioavailability (Thorat and Dalvi, 2015). Specifically, orthorhombic forms, although less stable at high temperatures, exhibited greater stability in dispersion over time, which can influence the long-term solubility and efficacy of curcumin formulations. Moreover, both ultrasound intensity and the type of additives were shown to affect the crystalline structure of curcumin, shifting from monoclinic to orthorhombic forms depending on precipitations conditions. Notably, amorphous curcumin nanoparticles exhibit improved apparent solubility and faster dissolution compared to crystalline counterparts, although they are generally less stable over time (Thorat and Dalvi, 2015). However, this disordered structure, while beneficial for bioavailability, often results in reduced physical stability, posing a challenge for long-term storage and practical application (Thorat and Dalvi, 2015). A key destabilization mechanism is Ostwald ripening, where smaller particles dissolve and redeposit onto larger ones, leading to particle growth and compromising the benefits of nano-sizing (Thorat and Dalvi, 2015; Panagiotou et al., 2009; Zhang et al., 2022). Notably, recent work showed that amorphous nanoparticles undergo rapid ripening within minutes, whereas crystalline particles remain stable for weeks due to a kinetic barrier, which highlights the critical role of particle structure in determining long-term stability (Behrens et al., 2025). Thus, precise control of nucleation and growth, along with a clear understanding of destabilization mechanisms, is essential for developing robust nanoformulations. In the nanoprecipitation process, rapid mixing of organic and aqueous phases induces solute supersaturation, followed by nucleation and subsequent particle growth, mainly *via* molecular diffusion of solute towards existing nuclei and, in some cases, particle aggregation. Among the factors influencing destabilization, solubility of the dispersed phase in the continuous medium is critical. Several studies have investigated Ostwald ripening in nanoparticles systems. For instance, Liu et al. (2007) demonstrated in β-carotene nanoparticles that smaller particles dissolve and redistribute mass to larger ones, with good agreement between experimental data and LSW theory (Liu et al., 2007). Similarly, Goubault et al. (2021) investigated Ostwald ripening in organic nanoparticle systems and showed that the irreversible adsorption of alkyl-coated nanoparticles at the liquid-liquid interface limits droplet coalescence, thereby slowing the ripening process (Goubault et al., 2021). These findings underscore the potential of nanoparticle-mediated stabilization mechanisms, reinforcing the importance of understanding and controlling these phenomena to improve nanoformulation stability. In a related study, Yadav and Kumar (2014) employed antisolvent precipitation for curcumin nanonization, a simple and cost-effective technique to optimize its physicochemical properties. Their work yielded curcumin nanoparticles (70–220 nm) with narrow size distribution and spherical morphology, and the size reduction markedly increased the *in vitro* release rate, thereby enhancing solubility and potential bioavailability compared to conventional curcumin powder (Yadav and Kumar, 2014). Key factors influencing particle size included the concentration of curcumin in solution and the proportion of antisolvent, with higher supersaturation levels favoring smaller particle formation. Their study also evaluated the stability of freeze-dried curcumin nanoparticles, showing that they retained their physical properties and release profiles over six months, particularly under controlled storage temperatures (2–8 °C and ambient conditions). Overall, these findings provide valuable insights into curcumin nanoformulation, underscoring their potential to enhance solubility and stability and supporting their broader applicability in functional formulations (Yuan et al., 2021; Chen et al., 2023). Formulation and characterization of curcumin nanoparticles have been widely studied, but their long-term stability remains insufficiently explored. Destabilization mechanisms such as aggregation, crystallization, and Ostwald ripening are not fully clarified. Concerning the stability of nanoprecipitated curcumin particles, to the best of our knowledge, no articles focused on a deep study of the mechanisms involved and the majority of the articles present the addition of other components (polysaccharide, surfactant, polymers) to enhance the nanoparticles stability without really understanding the mechanisms at work (Yadav and Kumar, 2014; Thorat and Dalvi, 2012; Chow et al., 2015). No articles report specific study of the destabilization mechanisms of nanoprecipitated curcumin particles.

In addition to modelling particle growth over time, particularly using the LSW model, to our knowledge, no study has examined the evolution of curcumin nanoparticle stability when the composition of the continuous phase is modified. Indeed, a critical factor influencing these processes is the solubility of the dispersed phase in the continuous medium, as variations in solubility can either enhance or suppress Ostwald ripening. The variation of this parameter, which plays a major role in the Ostwald ripening mechanism, allows this study to validate, in addition to the modelling of experimental data, that the destabilization of curcumin nanoparticles does indeed follow an Ostwald ripening mechanism. To explore this aspect, acetone content was systematically varied prior to nanoparticle formation. This study aims to identify and model the main destabilization mechanism of curcumin nanoparticles produced by nanoprecipitation, by systematically investigating common destabilization pathways, namely aggregation, coalescence, crystallization and Ostwald ripening. To achieve this, complementary and widely accessible techniques such as ^1^H NMR, XRD, DLS, HPLC and SEM were employed to probe particle size, solubility, morphology, and structural organization. The proposed methodology provides a framework that could be extended to other colloidal systems to better understand and control long-term stability without relying on stabilizing agents or encapsulation.

## 2 Materials & methods

### 2.1 Materials

Curcumin ≥65 % w/w (Cur) was purchased from Sigma Aldrich (France). Acetone was purchased from Carlo Erba (France). It was selected as solvent due to its strong solubilization capacity for curcumin, full miscibility with water, and ease of removal after processing. In addition, its low viscosity (0.3 mPa s at 20 °C) compared to other common solvents such as ethanol (1.2 mPa s at 20 °C) or DMSO (2.0 mPa s at 20 °C) favors rapid diffusion during nanoprecipitation which improves particle size homogeneity (Bovone et al., 2022). This choice is also supported by Yadav and Kumar (2014) who tested several solvents and found that acetone produced the smallest and most uniform curcumin nanoparticles.

### 2.2 Formation of curcumin nanoparticles using the antisolvent nanoprecipitation technique

Due to the hydrophobic nature of curcumin, it is initially dissolved in acetone to ensure complete solubilization before nanoprecipitation. Using the antisolvent nanoprecipitation technique, 500 μL of a 10 mg mL^−1^ curcumin solution in acetone was added all at once under stirring (250 rpm) over 10 s using a micropipette to 25 mL of ultrapure water leading to final acetone concentrations of 2 %, 4 %, or 6 % v/v, following the protocol adapted by Yadav and Kumar (2014).

To investigate the effect of solvent composition, nanoparticles were prepared in water/acetone mixtures adjusted to final acetone concentration of 2 %, 4 % or 6 % (v/v). For 0 % of acetone condition, nanoparticles were initially formed in a medium with a final acetone concentration of 2 % (v/v). The dispersion was then subjected to mild centrifugation at 4300 g for 10 min at room temperature, to concentrate them without inducing sedimentation. The supernatant was removed and replaced with ultrapure water, resulting in a predominantly aqueous medium.

### 2.3 Characterization of curcumin nanoparticles

#### 2.3.1 Antioxidant activity

The antioxidant activity of curcumin nanoparticles and curcumin powder was assessed using the ABTS radical scavenging assay, adapted from Vuillemin et al. (2020) (Vuillemin et al., 2020). A 7 mM ABTS solution and a 2.45 mM potassium persulfate solution were prepared in ultrapure water, mixed at 1:1 vol ratio and left to react overnight under stirring (500 rpm) in the dark at room temperature. Samples of curcumin powder and curcumin nanoparticles were dispersed in ultrapure water at different concentrations. A control without sample was also prepared. For each assay, 10 μL of sample dispersion was mixed with 1 mL of ABTS^•+^ solution. The mixture was left to react for 15 min at room temperature in the dark and absorbance was measured at 734 nm using 1 cm path length plastic cuvettes, after blank correction with ultrapure water. The radical scavenging activity (%) was calculated according to:

(1) $${A\text{BTS}\;}^{\cdot +}\text{scavening}\;\text{activity}\;\left(\%\right)=1-\left(\frac{{A\text{bs}}_{s\text{ample}}}{{A\text{bs}}_{c\text{ontrol}}}\right)\times 100$$

The results were expressed as IC_50_ values, defined as the sample concentration required to scavenge 50 % of ABTS^•+^ radicals. All measurements were performed in triplicate.

#### 2.3.2 Stability measurement

The stability of particle dispersions was assessed using a Turbiscan Lab Expert (Formulaction, France). Immediately after nanoprecipitation (or after centrifugation and redispersion for the 0 % condition), 20 mL of sample was transferred into a glass vial designed for Turbiscan analysis and placed in the instrument. Measurements were performed at 25 °C over 24 h using near-infrared pulsed light (λ = 800 nm).

Both transmission (T, light passing through the sample) and backscattering (BS, light scattered backward by the sample) were recorded as a function of sample height. Profiles were collected every 10 min during the first hour and then every hour up to 24 h. Overall dispersion stability was quantified using the Turbiscan Stability Index (TSI), which integrated T and BS variations along the sample height. Lower values indicated more stable dispersions, while higher values reflected destabilization phenomena such as aggregation, sedimentation, or creaming. TSI values were calculated at each time point and plotted as function of time to monitor stability evolution. All measurements were performed in triplicate to ensure reproducibility, allowing quantitative comparison of formulations prepared under different acetone concentrations.

#### 2.3.3 Size and polydispersity index measurements

The size evolution of curcumin nanoparticles was monitored over time using Dynamic Light Scattering (DLS). For each measurement, 1 mL of dispersion was transferred into a glass cuvette. Analyses were performed with a Zetasizer Nano-ZS instrument (Malvern Panalytical, UK) equipped with a He/Ne ion laser (λ = 532 nm), and data were collected at 173° on a backscattering detector. The average hydrodynamic diameter (Z-average) and polydispersity index (PdI) were calculated from the autocorrelation function of scattered light intensity, assuming spherical particles, at 25 °C. All measurements were carried out in duplicate.

#### 2.3.4 Modeling of particle size evolution

The growth of curcumin nanoparticles was modeled according to LSW theory which describes Ostwald ripening (Lifshitz and Slyozov, 1961; Wagner, 1961). The mean particle radius r(t) evolves with time as:

(2) $${r\left(t\right)}^{3}-r{\left(0\right)}^{3}=k\times t$$

where r(t) is the mean particle size at time t, r(0) is the initial mean particle size (t = 0), and k was a constant related to the physical parameters of the system, including interfacial tension, solubility, and the diffusion coefficient of the molecule in the continuous phase.

In this model, the ripening rate constant k was determined, reflecting key physical parameters governing Ostwald ripening. It was expressed as:

(3) $$k=\frac{8\psi v^{2}{\text{Dc}}^{\infty }}{9\text{RT}}$$

where ψ was the interfacial energy (J.m^−2^), v the molar volume of curcumin (m^3^.mol^−1^), D the diffusion coefficient of the curcumin in water (m^2^.s^−1^),C^∞^ the equilibrium solubility (mol.m^−3^), R the gas constant (J.mol^−1^.K^−1^), and T was the absolute temperature (K).

In this study, C^∞^ was measured in the presence of excess curcumin solid. Since no direct experimental value of D was available, an average diffusion coefficient (D = 21.5 μm^2^ s^−1^) was estimated from reported values of catechin and epicatechin in water at 25 °C (Srinivas et al., 2011). The molar volume of curcumin (v = 287.9 cm^3^ mol^−1^) was calculated from its molecular structure and density (McClements, 2018). The interfacial energy was assumed to be 0.001 J m^−2^, consistent with previous molecular dynamics simulations of curcumin in aqueous systems (Hazra et al., 2014).

By fitting the experimental data of r(t)^3^ – r(0)^3^ versus time, the constant k was obtained. Using equation (3) with known or estimated values of ψ, v, D, R, and T, the equilibrium solubility c∞ was calculated as:

(4) $$C^{\infty }=\frac{9\text{RTk}}{8{\psi v}^{2}D}$$

#### 2.3.5 Determination of the solubility and chemical stability of curcumin

Curcumin concentration in the aqueous phase was quantified by reverse-phase high-performance liquid chromatography (HPLC) using a Shimadzu system (Kyoto, Japan) with a GRACE Appolo C18 column (150 x 4,5 mm, 5 μm). An isocratic elution was performed with acetonitrile/water (50:50, v/v) at 1 mL min^−1^ and 25 °C. Curcumin was detected at 425 nm with a retention time of ∼12 min.

For solubility measurements, native curcumin powder was added in excess to ultrapure water or water/acetone mixtures (2,4, or 6 % v/v). After shaking at room temperature, samples were centrifuged (4300 *g*, 10 min) and the supernatant was analyzed by HPLC. The procedure was conducted in triplicate. In addition, dispersions obtained by nanoprecipitation were analyzed and compared with acetone solubilized curcumin to access potential change in chemical stability (UV-vis absorption spectra).

#### 2.3.6 Morphological analysis by Scanning Electron Microscopy (SEM)

The obtained curcumin nanoparticles were analyzed by scanning electron microscopy (SEM) to study their morphology. Samples were collected from the 2 % acetone medium at different times to observe the evolution of particle morphology over time. A drop of the particle dispersion was deposited onto aluminium stubs and dried at room temperature for 48 h. After deposition, the samples were coated with a thin layer of carbon to improve sample conductivity. Images were acquired using a Hitachi S-4800 SEM at an acceleration voltage of 1.0 kV. Observations were performed at various magnifications to provide an overview of the particles as well as detailed insights into their size and morphological distribution. Sizes were been obtained from numerous particles using ImageJ software.

#### 2.3.7 Nuclear Magnetic Resonance (NMR) analysis

Curcumin samples were analyzed by proton nuclear magnetic resonance (^1^H NMR) spectroscopy at 300 K using a Bruker Avance III HD 400 MHz spectrometer (Bruker BioSpin GmbH, Rheinstetten, Germany) equipped with a 5 mm BBFO probe with Z-gradient coils. Standard 5 mm NMR tubes were used. Two types of samples were prepared a curcumin solution at the final analysis concentration and a curcumin nanoparticle obtained by nanoprecipitation (acetone solution of curcumin added to ultrapure water), followed by resolubilization in acetone-d6 at a 1:2 dilution ratio. This resolubilization allowed the curcumin to fully dissolve and ensured that the final concentration matched that of the curcumin solution sample. NMR experiments were carried out using standard 1D proton (zg30) and water suppression (noesygppr1d) pulse sequences. The number of scans was 32, with a relaxation delay (d1) of 2 s, and a spectral width (sw) of 20 ppm. Data were processed using TopSpin 4.5.0 software (Bruker BioSpin GmbH, Germany).

#### 2.3.8 Crystallization determination by XRD

X-ray diffraction measurements were performed using a D8 Discover diffractometer (Bruker, Germany). Following nanoprecipitation (as described above), the curcumin nanoparticles were concentrated by centrifugation at 4300*g* for 10 min using a benchtop centrifuge. Individual samples were withdrawn at defined time points each measurement corresponded to one distinct sampling, which was immediately deposited for XRD analysis. The samples were measured on a zero-background holder (silicon wafer). Diffraction patterns were acquired with a scanning time of approximately 5 min per measurement, with samples collected every 10 min over a period of 1–2 h to monitor phase evolution (to determine whether the particles were amorphous or crystalline). The diffraction data were recorded over an angular range of 8°–24° (2θ), which was consistent with the main diffraction peaks reported for crystalline curcumin in the literature (Yadav and Kumar, 2014; Thorat and Dalvi, 2015), and processed by applying baseline correction and background subtraction using Diffrac Eva V2.1 software.

### 2.4 Statistical analysis

Statistical analyses were performed in R (version 4.2.2) using one-way ANOVA to compare the evolution of particle size under different solvent concentrations. A Tukey's post-hoc test was applied to identify differences between groups. The effect of time on the polydispersity index (PdI) was assessed using repeated-measures ANOVA to evaluate whether a trend was observed. Finally, the solubility of curcumin under different experimental conditions was compared using a Student's t-test, to evaluate differences between two conditions. All statistical analyses were performed with a significance threshold of p < 0.05.

## 3 Results & discussion

### 3.1 Antioxidant activity of curcumin nanoparticles

Curcumin is well known for its strong antioxidant activity due to its polyphenolic structure. In the present study, the antioxidant activity of curcumin nanoparticles was compared to that of native curcumin powder using the ABTS^•+^ radical scavenging assay. The IC_50_ value of curcumin powder was found to be 2964 ± 43 μg mL^−1^, consistent with values previously reported in the literature. In contrast, curcumin nanoparticles exhibited an IC_50_ of 3 ± 0.3 μg mL^−1^ indicating a difference in radical scavenging capacity between the two forms. This trend is consistent with the findings of Quirós-Fallas et al. (2022), who reported that encapsulation of curcumin into hybrid lipid-polymeric nanoparticles decreased the IC_50_ from 2444.8 μg mL^−1^ (free curcumin in water) to 9.55 μg mL^−1^ (Quirós-Fallas et al., 2022). Notably, the amorphous curcumin nanoparticles, prepared by a simple antisolvent precipitation without stabilizers, achieved an even lower IC_50_, suggesting improved dispersibility and surface reactivity compared to more complex carrier. This variation can be attributed to differences in solubility and dispersibility, as nanoparticles formation improves the apparent surface area available for radical interaction. Although only assessed *in vitro*, the lower IC_50_ observed for curcumin nanoparticles highlights their potential for improved functional performance compared to native curcumin powder, supporting their relevance as carriers for poorly soluble bioactives. As antioxidant activity depends not only on the intrinsic properties of curcumin but also on the stability of the colloidal system, it was essential to investigate the mechanisms governing nanoparticles evolution over time. Indeed, the antioxidant power of curcumin is enhanced at the nanoscale, so it seems important to maintain a stable dispersion whose size does not change over time in order to preserve maximum antioxidant capacity.

### 3.2 Curcumin NPs stability upon time

**Fig. 1:**
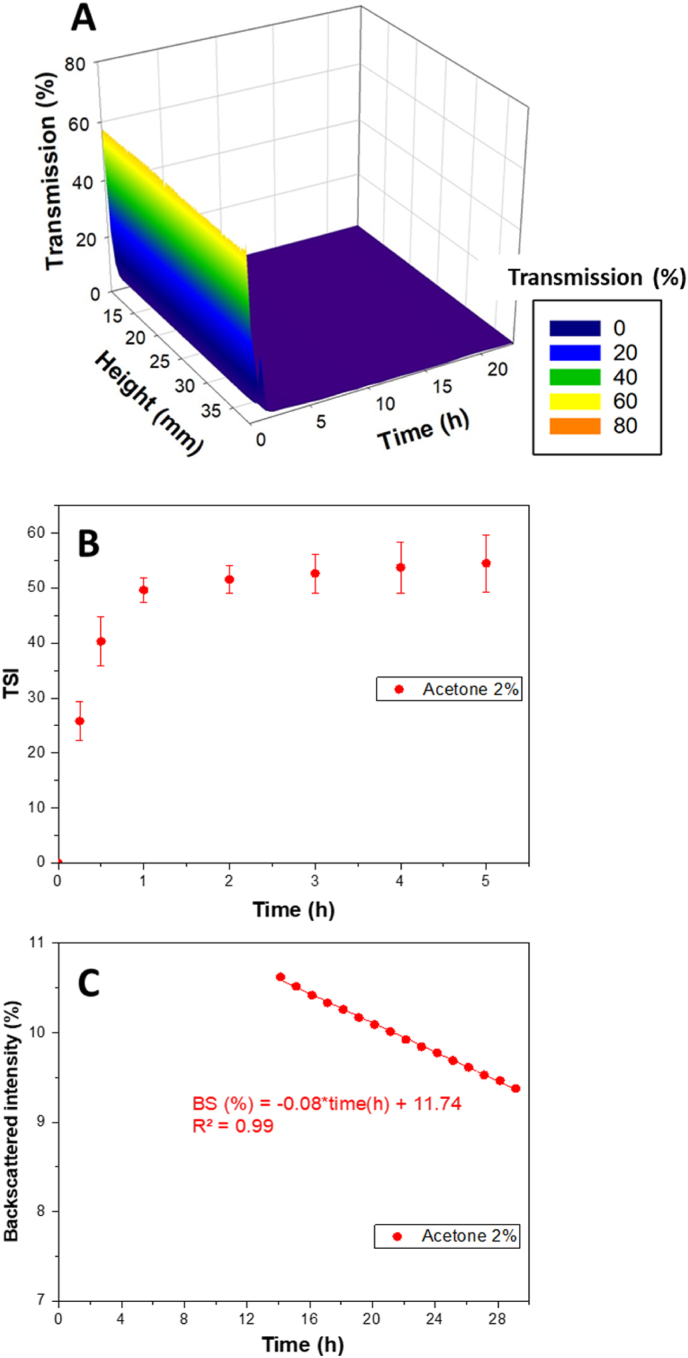
Evolution of light transmission (A), TSI values (B) and backscattering (C) of curcumin nanoparticle dispersions (2 % of acetone in the aqueous phase (v/v)).

The colloidal stability of the nanoparticles was investigated using static multiple light scattering (SMLS) analysis to monitor the stability of curcumin nanoparticles over time. Transmission and backscattering profiles were recorded over 24 h (Fig. 1).

The transmission profile revealed a rapid and sustained decrease within the first 2 h, followed by a plateau at near-zero transmission values that persisted for the remainder of the experiment (Fig. 1A) This indicated fast particle growth and persistent turbidity, without any visible clarification of the upper part of the sample. The absence of late-stage transmission increases typically associated with sedimentation suggested that no significant particle settling occurred during this time frame.

To complement the transmission analysis, the turbiscan stability index (TSI) was calculated over the entire height of the tube, since the signals remained homogeneous along the sample height, and analyzed over time (Fig. 1B). After 30 min, the TSI values exceeded 35 indicating significantly destabilization over time. However, TSI variations suggested that TSI alone was insufficient to distinguish destabilization effects. While the TSI provided a global indicator of system instability, it did not allow for a detailed assessment of how scattering properties evolve over time. Since backscattering remained homogeneous across the entire tube height, its evolution was analyzed over time to better understand the destabilization process.

To better characterize the destabilization process observed, backscattering evolution was analyzed in regions where the transmission dropped below 0.2 % (Fig. 1C). In the highly turbid zone, intense and stable scattering enabled the acquisition of well-defined backscattering profiles over time. Variations in backscattering intensity were spatially distributed across a large portion of the tube, indicating a gradual and homogeneous evolution of the system. The linearity of the signal change over time allowed extracted of the backscattering slope (BS%/h), which served as a proxy for the rate of structural rearrangement within the nanoparticles suspension. This parameter has previously been correlated with colloidal instability and particle reorganization in nanosystems lacking sufficient steric or electrostatic stabilization. The use of BS slope thus complemented TSI data by providing a localized and kinetic insight into destabilization dynamics. The increase in turbidity overtime suggests a growth in particle size, eventually leading to sedimentation.

**Fig. 2:**
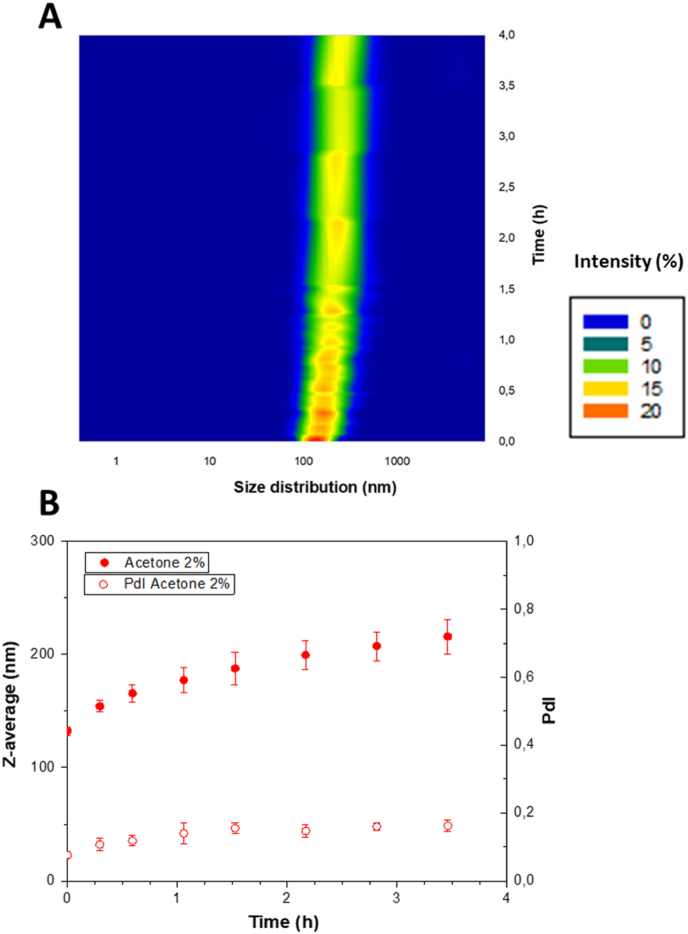
Evolution of size distribution over time (A) and particle size and PDI trends (B) of curcumin nanoparticle dispersions (2 % of acetone in the aqueous phase (v/v)).

To confirm that destabilization observed by SMLS was primarily related to particle growth rather than coalescence, aggregation, crystallization or Ostwald ripening, dynamic light scattering (DLS) measurements were performed during the first 4 h after nanoprecipitation. This period corresponded to the phase most significant transmission changes identified by SMLS analysis (Fig. 1.). The intensity-based particle size distribution (Fig. 2A) revealed a gradual shift of the main peak toward larger diameter, while maintaining a single distribution mode, suggesting continuous and homogeneous particle growth.

Z-average values (Fig. 2B) increased from 130 nm (±4) to 223 (±13) over the 4-h period, confirming size evolution over time. Despite this growth, the polydispersity index remained below 0.2, indicating the absence of broad size dispersion typically associated with aggregation.

To further rule out aggregation, ζ-potential measurements were carried out immediately after nanoprecipitation and again at the end of the 4-h observation period. In both cases, the ζ-potential remained below −30 mV, a threshold commonly considered sufficient to ensure electrostatic stabilization and prevent particle aggregation due to strong repulsive forces (Öztürk et al., 2024). Taken together, these observations supported the hypothesis that the observed destabilization was predominantly driven by molecular reorganization processes rather than aggregation.

These results were in line with commonly reported stability criteria for colloidal systems. In DLS analyses a PdI below 0.2 typically reflects a monodisperse population and indicates the absence of aggregation, which is usually accompanied by a marked broading of the size distribution (Danaei et al., 2018). Several studies have shown that an increase in particle size associated with a stable PdI often arose from slow structural rearrangement rather than true aggregation phenomena (Mora-Huertas et al., 2010; Peltonen and Hirvonen, 2010).

Likewise, ζ-potential values with an absolute magnitude greater than 30 mV are generally considered sufficient to provide electrostatic repulsion between particles, preventing aggregation even over extended time periods (Honary and Zahir, 2013; Bhattacharjee, 2016). In contrast, systems with |ζ| values below this threshold are more prone to flocculation due to insufficient repulsive forces. The persistence of ζ-potentials above 30 mV throughout the experiment therefore strongly supported the absence of significant aggregation in the present system.

While aggregation was excluded as primary destabilization mechanism based on ζ-potential and size distribution data, and sedimentation appeared limited during the initial hours, other processes such as crystallization, Ostwald ripening or coalescence could still contribute to observed particle growth. Clarifying these possibilities requires further investigation into the physical and structural characteristics of nanoparticles immediately after formation.

### 3.3 Physical and structural characterization of nanoparticles

#### 3.3.1 Determination of the physical state using NMR

The modulation of particle size over time combined with the exclusion of aggregation based on ζ-potential and DLS measurements suggested that another destabilization mechanism was involved. However, the possibility of coalescence could not be ruled without clarifying the physical state of the nanoparticles. Solid particles are typically not prone to coalescence, whereas liquid-like structures can merge over time. To investigate this point, NMR spectroscopy was used to determine the molecular mobility and physical state of curcumin within the colloidal system.

This technique was particularly suitable for distinguishing between solid and dissolved states, since NMR signal intensity and resolution are highly dependent on the molecular mobility. The greater the molecular mobility, the sharper and more intense the peaks. In contrast, molecules in the solid state exhibit limited mobility, leading to broadened or even undetectable signals.

In this study, curcumin nanoparticles synthesized *via* nanoprecipitation were directly analyzed by ^1^H NMR without prior dissolution. The resulting spectrum showed no detectable curcumin signals, indicating that curcumin in the nanoparticulate dispersion was not in a dissolved or highly mobile form. Upon addition of acetone-d6, clear and sharp signals corresponding to curcumin were observed, confirming its solubilization. These results confirmed that curcumin nanoparticles existed in a solid-like state immediately after formation. This observation is consistent with previous studies showing that antisolvent precipitation tends to favor the formation of amorphous or partially crystalline nanoparticles, which in turn influences their dissolution rate and overall stability (Yadav and Kumar, 2014).

**Fig. 3:**
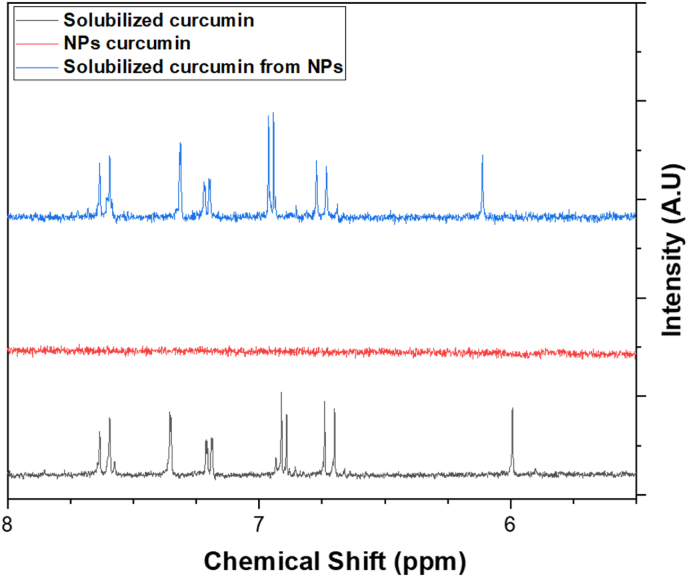
Comparison of ^1^H NMR spectra to assess molecular mobility: curcumin solubilized in acetone-d6 (black), curcumin nanoparticles (red), and resolubilized nanoparticles in acetone-d6 (blue).

The NMR spectra presented in Fig. 3 provide direct evidence of the physical state of curcumin nanoparticles. In the control solution, where curcumin was dissolved in acetone-d_6_ (0.1 mg/mL, black spectrum), characteristic peaks appeared: aromatic protons between 6.0 and 8.0 ppm. These well-defined peaks confirmed that curcumin was fully dissolved in this solvent. In contrast, the nano-dispersion of curcumin (0.2 mg/mL, blue spectrum) showed no detectable NMR signal, indicating restricted molecular mobility, a typical characteristic of solid-state systems (El and Sebakhy, 2021). Upon dilution of the nano-dispersion with acetone-d_6_ (0.1 mg/mL, red spectrum), characteristic curcumin signals reappeared, confirming that curcumin was present and could be solubilized. The absence of detectable NMR signals in the native nanoparticle dispersion supports the hypothesis that curcumin was initially present in a solid state, characterized by restricted molecular mobility, rather than in a freely mobile or dissolved form.

The physical state of nanoparticles played a crucial role in their stability. If curcumin nanoparticles existed in a liquid-like state, increased molecular mobility would facilitate material exchange between particles, accelerating the Ostwald ripening process and leading to a gradual increase in particle size. However, their solid state limited these effects, as studies have shown that solid formulations were more stable than liquid suspensions (Yadav and Kumar, 2014). Additionally, the precipitation method used can influence particle crystallinity. For example, in supercritical processes, nanoparticles with lower crystallinity exhibited faster dissolution rates (Rezaee et al., 2024).

Finally, although identifying the solid-state nature of curcumin nanoparticles was essential, their stability was likely influenced by the specific structural organization of the solid phase whether the nanoparticles were amorphous or crystalline. Structural organization must also be considered, as it directly affected solubility and dissolution kinetics. While confirming the solid-state nature of the nanoparticles excluded coalescence, further structural characterization was necessary to determine whether the particles remained amorphous or underwent crystallization over time.

#### 3.3.2 Structural organization analysis by XRD

While confirming the solid-state nature of curcumin nanoparticles is an important step, stability and dissolution behavior also depend on their structural organization whether they remain amorphous or transition to a crystalline state over time. X-ray diffraction (XRD) was therefore used to track the evolution of nanoparticle crystallinity in the first few hours following nanoprecipitation.

**Fig. 4:**
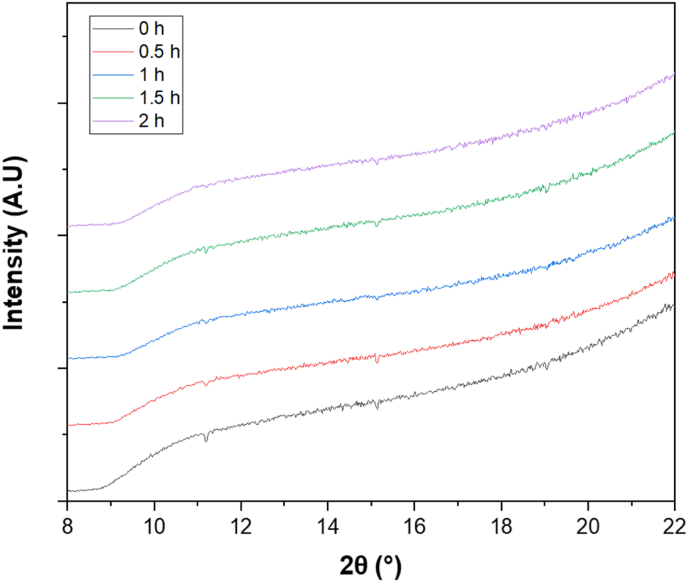
XRD analysis of curcumin dispersions over time: evaluation of crystallinity during the first 2 h. Spectra correspond to nanoparticles at 0 h (black), 0.5 h (red), 1 h (blue), 1.5 h (green), and 2 h (purple).

The X ray diffractograms, displaying diffraction intensity as a function of 2θ angle and time (Fig. 4), revealed that no distinct diffraction peaks were present during the first 2 h after nanoprecipitation. This indicated that the nanoparticles remained in an amorphous state, with no long-range molecular order but retaining short-range molecular organization. The absence of crystalline peaks suggested that the rapid precipitation process led to a disordered molecular arrangement, a phenomenon commonly observed in nanoparticulate systems where fast solvent removal prevented molecular reorganization. This was consistent with findings from Thorat and Dalvi (2014), who demonstrated that curcumin nanoparticles precipitated *via* the liquid antisolvent technique in the presence of ultrasound and stabilizers could remain in an amorphous state before transitioning to an orthorhombic crystalline form, depending on the additives used.

The fact that the amorphous nature persisted over 2 h suggested that crystallization did not occur spontaneously in the timeframe studied. This result contrasted with previous reports in which amorphous nanoparticles underwent a progressive transition to a crystalline state over time, depending on factors such as temperature, solvent composition, and residual moisture (Thorat and Dalvi, 2014). While the exact conditions for potential crystallization beyond the studied period remained to be explored, these findings provided key insights into the early-stage stability of curcumin nanoparticles and reinforced the idea that their dissolution behavior would be strongly influenced by their amorphous nature. In particular, it has been shown that amorphous formulations significantly enhanced the bioavailability of curcumin, as crystalline structures tend to exhibit poor water solubility (Urošević et al., 2022).

Additionally, alignment was observed with studies demonstrating that amorphization *via* advanced processing techniques could lead to a dramatic enhancement in the dissolution rate of curcumin, thereby increasing its apparent solubility and potentially improving its bioavailability in pharmaceutical formulations. Garbiec et al. (2025), showed that amorphization of curcumin using supercritical fluid technology led to a 300-fold increase in the apparent solubility compared to its crystalline form (Garbiec et al., 2025). This enhancement was attributed to the increased free-energy state of the amorphous form, which promoted dissolution and bioavailability. However, as noted in their study, maintaining the amorphous state was crucial, as spontaneous recrystallization could negate these benefits over time. Strategies such as incorporating stabilizing agents, such as hydrophilic polymers or co-formers like tryptophan, have been proposed to prolong the amorphous stability and prevent unwanted transitions back to the crystalline phase (Garbiec et al., 2025).

In addition to influencing solubility, the amorphous state of the nanoparticles could also impact their susceptibility to Ostwald ripening, as amorphous phases typically exhibited higher molecular mobility and solubility than their crystalline counterparts. The formation of nanoparticle aggregates and superstructures during the precipitation process, as reported by Thorat and Dalvi (2014), suggested that particle growth kinetics could play a critical role in long-term stability. Moreover, the ability of amorphous curcumin systems to remain supersaturated over extended periods, as demonstrated in studies using polymer-stabilized amorphous formulations, further supported the importance of controlling crystallization to maintain solubility and bioactivity (Garbiec et al., 2025). Overall, these results indicated that curcumin nanoparticles remained in an amorphous state during the early hours post-precipitation, ruling out crystallization as a contributor to the observed particle size increase.

### 3.4 Influence of acetone concentration on colloidal stability

Following the exclusion of aggregation (DLS and ζ), coalescence (^1^H NMR) or crystallization (XRD), the observed increase in particle size at 2 % acetone was attributed to Ostwald ripening. To further explore this hypothesis, the solubility of curcumin in the continuous phase was modulated by varying the acetone concentration (0 %, 4 % and 6 % v/v) as increased solubility is known to promote molecular diffusion and favor ripening process. SMLS and DLS analyses were conducted to evaluate the impact of acetone content on particle growth kinetics and overall colloidal stability.

**Fig. 5:**
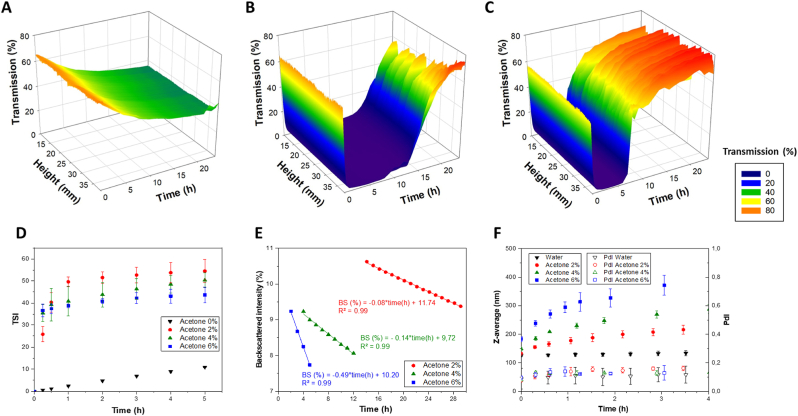
Evolution of light transmission (A: 0 %, B: 4 %, C: 6 % of acetone (v/v)), TSI values (D), backscattering (E), particle size and PDI trends in curcumin nanoparticle dispersions.

SMLS analysis was used to see the impact of acetone concentration on destabilization mechanisms and to evaluate whether the solubility of curcumin in the aqueous phase could influence particle growth and support the occurrence of Ostwald ripening. Transmission and backscattering profiles were recorded over 24 h for different acetone concentrations (0 %, 4 %, and 6 % v/v), as shown in Fig. 5A to 5C in addition to 2 % of acetone presented in Fig. 1A. Three successive phenomena can be identified from the transmission profiles. Initially, a decrease in transmission was observed, reflecting an increase in turbidity due to particle growth. This was followed by a plateau with near-zero transmission, indicative of a highly turbid medium in which particles continued to grow. Finally, a progressive increase in transmission was detected in the upper part of the tube, suggesting clarification of the medium, likely due to the sedimentation of larger particles. The presence and timing of these stages varied according to the acetone concentration. In the absence of acetone (Fig. 5A), transmission decreased slowly and uniformly across the tube, from about 60 % at the beginning of the experiment to approximately 40 % after 24 h, which suggested a steady and homogeneous growth of curcumin particles without sedimentation during this period. At higher acetone concentrations, namely 4 % and 6 % (Fig. 5B and 5C), a similar initial fall in transmission was observed, but it was followed, after several hours, by a gradual increase in transmission in the upper region of the tube, suggesting that particle growth was accompanied by sedimentation, resulting in a clarified supernatant. These results highlighted the influence of acetone on the kinetics of particle growth and destabilization. Increasing acetone concentrations likely enhance the solubility of curcumin at the initial time point, favoring faster growth rates and sedimentation processes that became apparent within the 24h window.

A more rapid increase in TSI (Fig. 5D) was observed in the different acetone/water mixtures compared to the pure water (0 % acetone), confirming faster destabilization with increasing acetone concentration. After 30 min, the TSI values exceeded 35 for 4 %, and 6 % acetone mixtures, respectively, whereas it remained at only 5 with the pure water, demonstrating significantly better stability in water compared to acetone/water mixtures. However, TSI variations between 2 %, 4 %, and 6 % acetone overlapped, suggesting that TSI alone was insufficient to distinguish destabilization effects at different acetone concentrations. These results were aligned with previous studies where SMLS analysis effectively identified aggregation and phase separation dynamics in colloidal suspensions, emphasizing TSI variability in different solvent conditions (Santonocito et al., 2020).

In Fig. 5E, for 0 % acetone condition, transmission never fell below 40 %, preventing significant backscattering analysis in this case. In contrast, with different acetone concentrations (4 %, and 6 %), intense scattering was observed, enabling clear backscattering profiles. However, the spatial window over which meaningful backscattering changes occurred became narrower as acetone concentration increased, suggesting a more localized and faster destabilization process. These changes, becoming more spatially confined as acetone increased, indicated that the system reached equilibrium more rapidly. At 6 %, signal variations were concentrated near the bottom, consistent with rapid particle growth followed by sedimentation. In the regions where T < 0.2 %, the backscattering slope (BS%/h) was extracted and found to increase with acetone concentration. The highest slope was observed at 6 % acetone, followed by 4 % and 2 %, indicating that higher acetone concentrations accelerate the evolution of the system. This suggested that the backscattering slope, which represented the rate of system evolution, was well correlated with nanoparticle destabilization rates, confirming its relevance as an indicator of particle reorganization.

Similar trends have been observed in nanoprecipitation-based systems, where instability increased when stabilizers were absent or insufficient (Kim et al., 2021). This suggested that the faster evolution observed at higher acetone concentration could be attributed to enhanced curcumin solubility in the continuous phase, which increased the driving force for Ostwald ripening. By modifying the saturation state of the system, acetone accelerated molecular diffusion and promoted faster reorganization of nanoparticles through dissolution and reprecipitation mechanism. The increase in transmission at later time points, particularly at 4 % and 6 % acetone, indicated a progressive clarification of the upper part of the sample, consistent with particle sedimentation resulting from particle growth induced by Ostwald ripening. This hypothesis was supported by previous studies showing that nanoparticle growth was driven by dissolution-reprecipitation processes, particularly in environments favoring high molecular mobility (Gommes, 2019; Behrens et al., 2025). The correlation between higher solubility and increased instability suggested that Ostwald ripening could be a dominant destabilization mechanism in the present system. A similar conclusion was reached in a study on stability of a nanosuspension of alkylated-dextran nanoparticles used to stabilize Pickering emulsions where the increase in transmission over time correlated with nanoparticle dissolution and reprecipitation mechanisms (Maingret et al., 2022). These findings underlined how nanoparticle stability was critically influenced by formulation parameters, whether in curcumin systems or in biodegradable Pickering emulsions, and suggested possible mechanisms that could be relevant for the present work.

As observed at 2 % acetone, evolution of particle size was further investigated at higher acetone concentrations to assess how solvent composition influenced growth kinetics, potentially consistent with an Ostwald ripening mechanism.

Z-average and the polydispersity index (PDI) were monitored as a function of acetone concentration over the same 4 h period (Fig. 5F). The evolution of the Z-average over the first 4 h confirmed that increasing acetone concentration significantly accelerated particle growth. At 0 % of acetone, the Z-average increased from 125 nm (±5) to 134 (±9) nm within 4 h, whereas at 2 % of acetone, it grew from 130 nm (±4) to 223 nm (±13) over the same period. In the presence of 4 % and 6 % of acetone, the particle size increased more rapidly, reaching 250 nm and 350 nm, respectively, after 4 h. These results quantitatively demonstrated that higher acetone concentrations enhanced particle growth dynamics. Previous studies on antisolvent precipitation, particularly those by Thorat and Dalvi (2012), have shown that such phenomena resulted from a rapid increase in supersaturation, leading to homogeneous nucleation followed by controlled particle growth predominantly through diffusion rather than aggregation. These findings aligned with the present observations, where acetone played a key role in modifying the thermodynamic equilibrium of the system, thereby accelerating particle growth dynamics. However, despite this increase in size, the polydispersity of the system remained relatively low across all tested conditions, with PdI values below 0.2. This suggested that during the initial phase (first minutes after mixing) particle growth occurred in a relatively homogeneous manner, without the formation of a highly heterogeneous population with widely dispersed sizes. These results contributed to understanding the nanonization of poorly soluble compounds by highlighting how antisolvent addition, such as water, to a curcumin-acetone solution impacts both particle size and stability. In the specific case of curcumin nanoparticles, the findings were consistent with recent advances in formulation strategies (Gayathri et al., 2023), and provide a useful basis for optimizing stabilization and controlling particle growth in colloidal systems.

### 3.5 Growth mechanisms (LSW analysis)

**Fig. 6:**
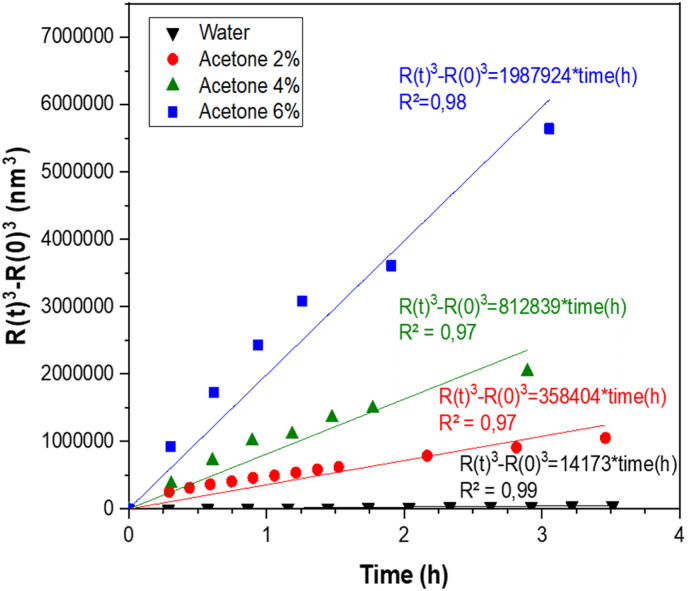
LSW-based modeling of growth kinetics in curcumin nanoparticle dispersions.

To determine whether the observed particle growth was consistent with Ostwald ripening, the LSW model was applied. Fig. 6 shows the evolution of R^3^ − R_0_^3^ over time. In all solvent conditions, a general linear trend was observed, suggesting that the process could be described by the LSW model.

However, as reported for other nanoparticle systems such as β-carotene dispersions, deviations from ideal linearity were also detected (Liu et al., 2007). These deviations likely arise from solvent interactions, initial particle size distribution and interfacial effects. Despite this, the determination coefficients remained high (R^2^ > 0.90), and the slope of the regression increased with acetone concentration, confirming that higher solvent content accelerated particle growth. Similar to previous findings (Liu et al., 2007), changes in solubility were the main driving force: while antisolvent addition reduced the ripening rate in β-carotene systems, acetone here increased curcumin solubility, enhancing dissolution reprecipitation and accelerating particle coarsening.

From the slopes, the ripening rate (K) was extracted and used to estimate the equilibrium solubility of curcumin (C^∞^) according to the LSW equation (3). The values obtained for each condition are reported in Table 1. As acetone concentration increased the calculated solubility also rose in agreement with HPLC measurements. This consistency demonstrates that solubility changes directly influenced the kinetics of particle growth and validates the relevance of the LSW approach for describing ripening in curcumin dispersion.

**Table 1:** The ripening rate constant (K) and curcumin solubility (c∞) estimated from the LSW model and obtained from HPLC measurements under different acetone concentrations.

Final concentration of acetone in water (% v/v)	K (m^3^.s^−1^)	C^∞^ by modeling (mg.L^−1^)	C^∞^ by HPLC (mg.L^−1^)
**0**	3.9 × 10^−27^ (±7.3 × 10^−29^)	2.32 (±0.02)	10.6 (±3.0)
**2**	10.0 × 10^−26^ (±6.0 × 10^−27^)	58.75 (±3.57)	38.0 (±2.1)
**4**	2.3 × 10^−25^ (±1.4 × 10^−26^)	133.25 (±8.24)	130.5 (±12.0)
**6**	5.5 × 10^−25^ (±3.1 × 10^−26^)	325.90 (±18.53)	196.1 (±23.2)

Nevertheless, deviations from ideal LSW predictions highlight the intrinsic limitations of the classical framework when applied to real nanoscale systems. The LSW theory assumes infinite dilution, negligible particle-particle interactions, and the absence of transient nucleation or structural rearrangement. As emphasized in comprehensive reviews of Ostwald ripening theories (Baldan, 2002), classical descriptions tend to oversimplify the early stages of nanoparticle evolution, where transient, non-classical growth pathways often dominate.

More recent theoretical approaches further illustrate these limitations. For example, stochastic or reaction-diffusion based models explicitly account for finite volume fractions, kinetic heterogeneities, and realistic particle-particle interactions, providing a more realistic description of nanoparticle coarsening (Zhu and Su, 2023). Zhu and Su (2023) show that nucleation growth dynamics involve a sequence of activated size-dependent steps that generate transient near equilibrium, stochastic fluctuations, and crossover regimes before the system ultimately converges toward asymptotic LSW-like behavior. These findings reinforce the view that early deviations from linearity are expected and do not contradict the occurrence of Ostwald ripening (Zhu and Su, 2023).

Altogether, these modern perspectives help clarify why deviations from ideal LSW behavior often appear in real systems. As highlighted in recent analyses of nucleation and growth pathways, early-stage dynamics can involve short-lived intermediates and stochastic fluctuations that fall outside the assumptions of classical theories (Zhu and Su, 2023; Jun et al., 2022). These early-stage effects do not rule out Ostwald ripening. Instead, they explain why experimental data may show slight non-linearities before approaching LSW-like behavior. The LSW model therefore remains suitable for identifying and quantifying the main ripening mechanisms in our system, as long as these expected early-stage deviations are taken into account.

In the present study, despite moderate departures from perfect linearity, the high determination coefficients and the consistent increase of the ripening constant with acetone concentration confirm that the LSW approximation remains appropriate for describing the dominant mechanism. Most importantly, the combination of the LSW analysis with this original experimental strategy, modulating the continuous phase composition to tune curcumin solubility, provides strong evidence that particle coarsening is governed by Ostwald ripening. The agreement between modeled and experimentally measured solubility values further validates this interpretation.

### 3.6 Morphological characterization of nanoparticles

**Fig. 7:**
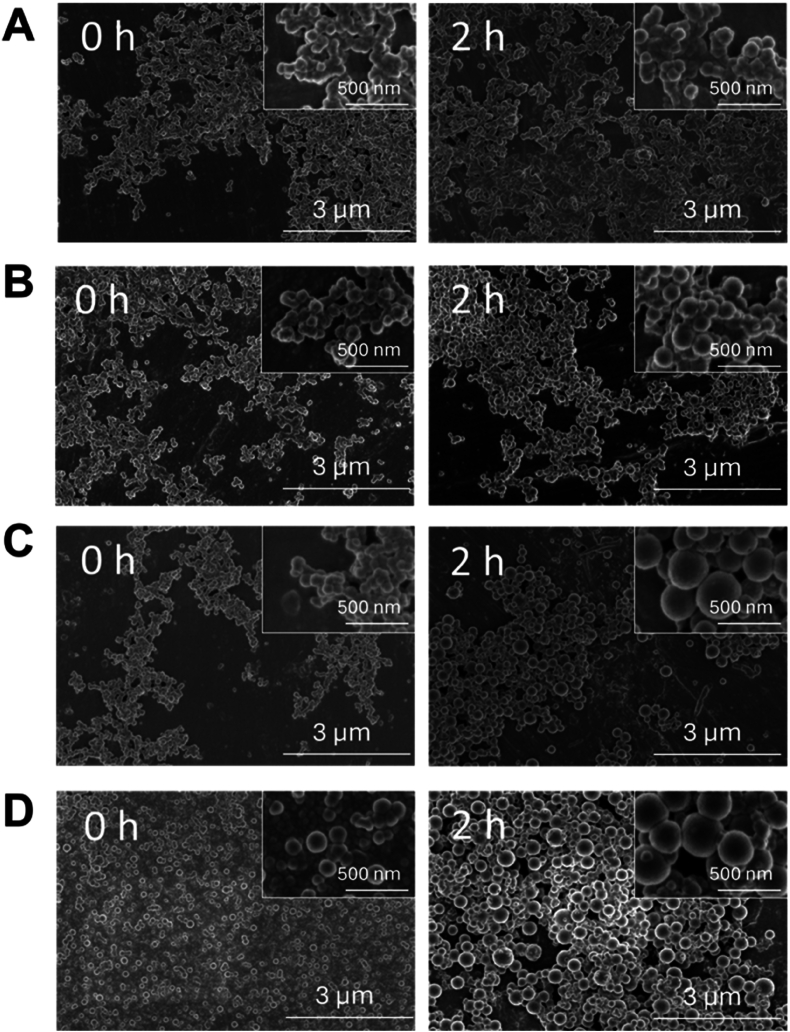
Impact of acetone concentration on the morphology of curcumin nanoparticles: SEM observations at 0 h and 2 h (A) water, (B) 2 %, (C) 4 %, (D) 6 % of acetone.

To further investigate the growth dynamics of curcumin nanoparticles, Scanning Electron Microscopy (SEM) was employed to analyze their morphology and size evolution over time. The results obtained are presented in Fig. 7, providing visual insights into the structural changes occurring during the process. At 0 h, SEM images confirmed that nanoparticles exhibited a spherical morphology, with sizes closely matching those obtained through DLS measurements. Specifically, the SEM analysis revealed an average particle size of 123 nm (±30) in water, 133 nm (±22) in 2 % acetone, 119 nm (±14) in 4 % acetone, and 147 nm (±28) in 6 % acetone. These values were in reasonable agreement with the DLS measurements, which reported initial Z-average sizes of 125 nm (±5) in water, 130 nm (±4) in 2 % acetone, 119 nm (±14) at 4 % and 147 nm (±28) 6 % acetone, likely due to hydration effects in DLS that increase the hydrodynamic diameter compared to dry-state SEM measurements. After 2 h of incubation, a significant increase in particle size was observed, consistent with the DLS analysis. SEM images showed that the average size increased to 144 nm (±27) in water, 169 nm (±34) in 2 % acetone, 265 nm (±85) in 4 % acetone, and 339 nm (±39) in 6 % acetone. In comparison, DLS results indicated particle sizes of 130 nm (±6) in water, 199 nm (±13) in 2 % acetone, 248 nm (±10) in 4 % acetone, and 327 nm (±32) in 6 % acetone, again reflecting the inclusion of the solvation layer in DLS measurements. The increase in size was more pronounced at higher acetone concentrations, corroborating the trend observed in DLS data and confirming that acetone promoted nanoparticles growth over time. Despite this growth, the particles remained relatively homogeneous in size and shape, as further confirmed by the low PDI values, indicating a uniform distribution of nanoparticles in solution. This homogeneous growth pattern suggested that the observed increase in size was not caused by aggregation or flocculation, which would typically led to a broader size distribution and more irregularly shaped clusters. Instead, it was consistent with an Ostwald ripening mechanism, as described in similar nanoparticle precipitation systems by (Thorat and Dalvi, 2012).

These results are in accordance with those reported by Gayathri et al. (2023) who reviewed various nano-formulation approaches for curcumin delivery (Gayathri et al., 2023). They emphasized that controlled nanoparticle growth, as observed in the present study, was key to enhancing bioavailability and therapeutic efficiency. The relatively stable dispersion of the prepared nanoparticles over time suggested that growth remained controlled, avoiding excessive broadening of the size distribution, which is a challenge in many nanoprecipitation systems.

## 4 Conclusion & perspectives

In this study, SMLS and DLS analyses revealed that curcumin nanoparticles progressively increased in size over time, eventually leading to sedimentation. ζ-potential measurements confirmed that aggregation was not the cause of destabilization, as values remained above the threshold for electrostatic stability. ^1^H NMR and XRD analyses demonstrated that the nanoparticles were in a solid yet amorphous state, thereby ruling out coalescence and crystallization as dominant mechanisms. Subsequently, modifying the composition of the continuous phase impacted curcumin solubility and resulted in different destabilization rates. This supported the hypothesis that Ostwald ripening was the prevailing destabilization process.

The results highlight that low polydispersity alone is not sufficient to prevent Ostwald ripening. In fact, as particles grow, sedimentation becomes increasingly likely, further compromising long-term stability. Several strategies can be envisaged to limit Ostwald ripening, such as maintaining nanoparticles in the aqueous phase, increasing the viscosity of the medium (e.g., by adding glycerol) to slow down molecular diffusion, or lowering the storage temperature to reduce solubility-driven mass transfer. In parallel, surface coating approaches using food-grade polymers such as chitosan or gum arabic could further stabilize the system by limiting solute exchange, enhancing colloidal stability, and preserving the amorphous structure.

From a formulation perspective, identifying Ostwald ripening as the main destabilization pathway provides a concrete basis for developing curcumin dispersions that remain stable under realistic food conditions. In particular, knowing that particle growth is driven by solubility enables rational strategies such as reducing solvent affinity, adjusting pH or ionic strength to minimize curcumin solubility, or applying food-grade polysaccharide coatings to limit molecular diffusion and show dissolution reprecipitation. These approaches are commonly used in food colloids and can now be targeted more effectively thanks to the mechanistic insight provided by this study.

Moreover, the nanoprecipitation strategy remains valuable since it improves the apparent solubility of curcumin while preserving its antioxidant potential, both of which are essential for its incorporation into functional food matrices. The exploration of greener solvents, such as natural deep eutectic solvents discussed by Kunz and colleagues, also represents a promising direction for developing fully food-compatible nanodispersions (Häckl and Kunz, 2018). Altogether, these findings highlight that understanding the destabilization mechanism is a key step toward designing curcumin nanoformulation adapted to real food environment.

## CRediT authorship contribution statement

Nicolas Didat: Conceptualization, investigation, formal analysis, visualization, writing and original draft.

Jordane Jasniewski: Supervision, project administration, writing, review & editing.

Pierrick Durand: Data curation (XRD), validation, writing, review & editing.

Sabine Bouguet-Bonnet: Data curation (NMR), investigation, writing, review & editing.

Younes Bouizi: Data curation (NMR), investigation, writing, review & editing.

Florentin Michaux: Methodology, supervision, writing, review & editing.

## Data availability statement

All data related to this article are available at this address: https://doi.org/10.57745/PGXRJM.

## Declaration of generative AI and AI-assisted technologies in the writing process

During the preparation of this work, the author used ChatGPT (OpenAI) to improve the clarity and language of some parts of the manuscript. The author reviewed and edited the content as needed and takes full responsibility for the final version.

## Funding

This research did not receive any specific grant from funding agencies in the public, commercial, or not-for-profit sectors.

## Declaration of competing interest

The authors declare that they have no known competing financial interests or personal relationships that could have appeared to influence the work reported in this paper.
